# Resistive Exercise for Arthritic Cartilage Health (REACH): A randomized double-blind, sham-exercise controlled trial

**DOI:** 10.1186/1471-2318-9-1

**Published:** 2009-01-13

**Authors:** Angela K Lange, Benedicte Vanwanseele, Nasim Foroughi, Michael K Baker, Ronald Shnier, Richard M Smith, Maria A Fiatarone Singh

**Affiliations:** 1 Exercise, Health and Performance Faculty Research Group, Faculty of Health Sciences, University of Sydney, Sydney, NSW, Australia; 2 Symbion Clinical Research Imaging Centre, Sydney, NSW, Australia; 3 Faculty of Medicine, University of Sydney, Sydney, NSW, Australia; 4 Hebrew SeniorLife and Jean Mayer USDA Human Nutrition Center on Aging at Tufts University, Boston, MA, USA

## Abstract

**Background:**

This article provides the rationale and methodology, of the first randomised controlled trial to our knowledge designed to assess the efficacy of progressive resistance training on cartilage morphology in women with knee osteoarthritis.

Development and progression of osteoarthritis is multifactorial, with obesity, quadriceps weakness, joint malalignment, and abnormal mechanical joint forces particularly relevant to this study. Progressive resistance training has been reported to improve pain and disability in osteoarthritic cohorts. However, the disease-modifying potential of progressive resistance training for the articular cartilage degeneration characteristic of osteoarthritis is unknown. Our aim was to investigate the effect of high intensity progressive resistance training on articular cartilage degeneration in women with knee osteoarthritis.

**Methods:**

Our cohort consisted of women over 40 years of age with primary knee osteoarthritis, according to the American College of Rheumatology clinical criteria. Primary outcome was blinded measurement of cartilage morphology via magnetic resonance imaging scan of the tibiofemoral joint. Secondary outcomes included walking endurance, balance, muscle strength, endurance, power, and velocity, body composition, pain, disability, depressive symptoms, and quality of life.

Participants were randomized into a supervised progressive resistance training or sham-exercise group. The progressive resistance training group trained muscles around the hip and knee at 80% of their peak strength and progressed 3% per session, 3 days per week for 6 months. The sham-exercise group completed all exercises except hip adduction, but without added resistance or progression. Outcomes were repeated at 3 and 6 months, except for the magnetic resonance imaging scan, which was only repeated at 6 months.

**Discussion:**

Our results will provide an evaluation of the disease-modifying potential of progressive resistance training for osteoarthritis.

**Trial Registration:**

ANZCTR Reference No. 12605000116628

## Background

### A rationale for implementing progressive resistance training as a disease-modifying treatment for knee osteoarthritis

Osteoarthritis (OA) is one of the most common musculoskeletal disorders in the world, affecting 9.6% of men and 18.0% of women ≥ 60 years of age worldwide [1]. In 1997, arthritis and other rheumatic conditions cost the United States (US) $86 billion dollars (equating to approximately 1% of the US gross domestic product) [2]. The development and progression of OA is multifactorial, with quadriceps weakness [3], joint malalignment [4], obesity [5], and abnormal mechanical joint forces [6] particularly relevant to this study.

### Is quadriceps weakness a cause or a consequence of knee OA?

Some studies conclude that quadriceps weakness is a possible preceding factor in the development of OA [7], while other studies support the possibility that quadriceps weakness contributes to disease progression [3]. Irrespective of the timing, once OA has developed, quadriceps weakness reduces the ability to decelerate leg movement and the leg is less able to absorb impulse loads transmitted through the tibiofemoral joint; leading to an acceleration of cartilage degeneration [3].

Medial compartment OA is associated with varus malalignment, and lateral compartment OA is associated with valgus malalignment [4]. A vicious cycle of malalignment then exists, whereby joint malalignment worsens the underlying excessive joint compartment forces (i.e. varus alignment increases the medial compartment load, and valgus alignment increases lateral compartment load in the knee during gait [8]) which contribute to OA progression in the respective compartments [4]. Knee malalignment is also considered to be a likely mediator of the OA-obesity relationship [8], as increased loading of malaligned, weakly supported joints accentuates the malalignment. Tendon stiffness is directly associated with muscle strength [9]; having stiffer tendons (less tendon elongation) improves the overall function and efficiency of the tendon as a stabilizer of the joint. By contrast, human studies have shown strengthening muscles (an adaptation to progressive resistance training (PRT)) increases stiffness and Young's Modulus in tendons around the knee joint which reduces the risk of tendon strain, and increases joint stability [9].

Muscle weakness may also play a role in the observed gender difference in the prevalence of OA, and the importance of obesity as a risk factor for OA. Firstly, radiographic OA is approximately twice as prevalent in women as it is in men [10]. Secondly, obesity is considered a risk factor for the development and progression of knee OA [8], and the association between obesity and OA is much stronger in women than in men [11]. The increased risk of knee OA associated with obesity appears to be related to mechanical rather than metabolic risk factors [8,12,13]. Women are weaker than men [14] and have lower muscle mass at all ages [15]. However, this gender disparity is diminished when lower extremity muscle strength is expressed per kilogram of muscle mass [16]. Reduced absolute and relative muscle mass in women means that they will be mechanically disadvantaged compared to men at the same absolute level of obesity or body mass index (BMI), as they have less muscle mass/strength to support the same body mass during standing and ambulation.

OA is considered a chronic degenerative disorder that is characterized by a loss of articular cartilage [17]. Due to the avascular nature of articular cartilage, cartilage development, maintenance and aging is dependent upon the type and magnitude of mechanical loading [18]. Immobilization or load deprivation alters the morphological, biochemical and biomechanical properties of articular cartilage [18], and ultimately results in decreases in cartilage thickness. More specifically, research has shown a significant 20% decrease in animal hyaline cartilage thickness of the femur following 11 weeks of immobilization [19]. By contrast, intermittent hydrostatic pressure during the early stages of OA in animals has been shown to maintain cartilage function [18]. In addition, animal studies have demonstrated a beneficial effect of moderate exercise on chondral lesion severity on induced (ACL-transected) OA [20], and strenuous wheel running exercise (6–12 km/day) is associated with normal cartilage (as opposed to fissuring, pitting, and fibrillation seen in the articular cartilage of sedentary hamsters who lived in individual housing without running wheels) [21]. This is supported by recent cross-sectional research revealing a higher tibial cartilage volume with both previous (baseline) and current (10 years after baseline assessment) participation in vigorous activity in healthy adults with no history of joint injury or trauma [22]. In addition, walking was also associated with a lower risk of bone marrow lesions [22]; which have previously been shown to be related to knee pain and cartilage degeneration [23,24].

### Specific rationale for resistance training exercise

**Figure 1:**
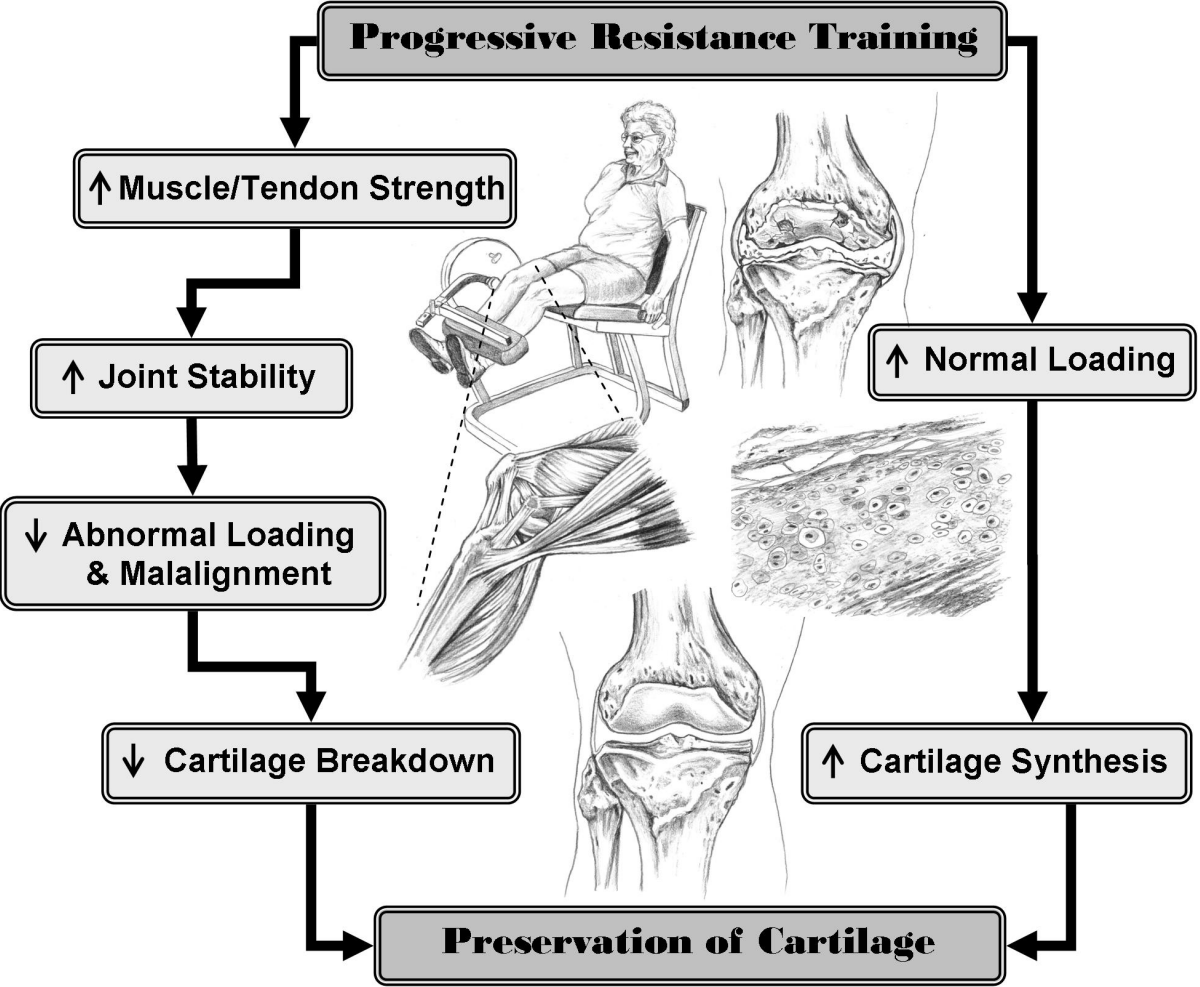
**Flow diagram detailing the rationale for implementing a progressive resistance training program for people with knee osteoarthritis**.

Resistance training or strength training as it is commonly known, has beneficial effects on cardiovascular disease, insulin action, bone density, energy metabolism, psychological health and functional status in various elderly populations [25,26]. To date, approximately 18 randomized controlled trials implementing a resistance training intervention in isolation have been conducted in knee OA populations. Improving pain and disability in OA cohorts is a common hypothesis and primary outcome in many of these studies. However, changes in the underlying pathophysiology of the articular cartilage have not been reported in any of these trials. Based on the animal and human data reviewed above, progressive resistance training (PRT) also has the potential to reduce OA progression through 2 proposed pathways (see Figure 1). Increased muscle strength and tendon stiffness secondary to PRT [9] facilitates a strong balanced co-contraction of knee extensor and flexor muscles, and may thus improve joint stability and reduce varus and valgus instability [27-29]. In the first pathway by improving knee joint stability, abnormal loading and harmful forces generated during walking may be reduced; helping to protect and prevent further cartilage degeneration [3,30]. In the second pathway, the moderate controlled loading of PRT may stimulate cartilage synthesis [31], offsetting or delaying the changes usually seen in OA.

The purpose of this article is to provide a detailed rationale and methodology of the Resistive Exercise for Arthritic Cartilage Health (REACH) trial. By publishing the detailed methodology prior to the final outcome publication limits the potential for publication bias.

## Methods

### Study Design and Setting

This study was a double-blind randomized, sham-exercise controlled clinical trial. Ethical approval was obtained from The University of Sydney Human Research Ethics Committee on the 13^th ^December 2004 (Reference No. 12-2004/2/7848) and written informed consent was obtained from all participants. This study has been lodged with the Australian New Zealand Clinical Trials Registry (ANZCTR Reference No. 12605000116628). The study was conducted at the Cumberland Campus of University of Sydney in Lidcombe NSW Australia. MRI Scans were performed at the Symbion Clinical Research Imaging Centre in Randwick NSW Australia.

### Eligibility Criteria

Women over 40 years of age in stable health with primary OA in at least 1 knee, according to the American College of Rheumatology clinical criteria [32] were included. Participants were excluded if they had secondary OA (i.e. OA diagnosed due to trauma, surgery, or other disease process); joint injury, injection or surgery within the past 6 months or knee joint replacement; already participated in structured exercise more than 1 day per week during the previous 3 months; any contraindications to exercise and/or magnetic resonance imaging (MRI); severe functional limitation or cognitive impairment.

### Recruitment

Participants were recruited from April 2005 to December 2006 from cohorts of previous research studies conducted at the University, articles and advertisements in local newspapers, information talks at local community and senior citizen centres, flyers in local businesses, and word-of-mouth.

### Medical Screening

A telephone screening questionnaire was followed by a physician history and physical exam in potential subjects.

### Intervention

Participants randomized to the intervention group performed resistance training at 80% of their peak strength using pneumatic resistance machines (K400 model, Keiser Sports Health, Inc, Fresno, CA, USA). The exercises included unilateral knee extension, standing hip abduction and adduction; and bilateral knee flexion, leg press, and plantarflexion. All exercises were performed for 3 sets of 8 repetitions (6–9 sec/repetition) with 10–15 seconds rest between repetitions and 1–2 min rest between sets. Maximum strength tests (1 repetition maximum or 1RM) were performed fortnightly and a new 80% load was prescribed; in between strength tests participants were prescribed 3% increments in load per session as tolerated. An intensity rating of 15–18 on the Borg Rating of Perceived Exertion (RPE) scale [33] was considered optimal and was used to adjust the load between 1RM measurements to assure the intended continuous progressive overload. Exercises and or resistance were modified daily according to participants' symptoms. Full range of motion was utilized unless limited by pain. In some participants, severe pain throughout the range of motion required substitution of isometric exercises of particular exercises intermittently during the 6 month of training.

The sham intervention was designed to closely replicate virtually all of the elements of the active exercise condition (modality, setting, supervision, equipment, volume, duration, frequency) with the notable exception of intensity, as we hypothesized that intensity would be the critical prescriptive element leading to robust adaptations in both proximal (muscle and tendon strength/hypertrophy) and distal outcomes (cartilage morphology) outcomes.

Participants randomized to the sham-exercise group trained on the same equipment as the intervention group except hip adduction, and performed knee extension bilaterally. Minimal resistance was set on the machine (weight of bar/foot plates only) and no progression was introduced. Exercise volume was reduced to 2 sets of 8 repetitions, using same speed as in the PRT group.

Both groups trained 3 times per week for 6 months under supervision of an exercise physiologist at the University in a ratio of 1:1–3. If sessions were missed due to illness or holidays, those sessions were added onto the end of the 6 month intervention. Up to 1 month extension was allowed to complete the 78 sessions (compliance was calculated as the percentage attended out of 78 sessions available).

### Adverse events

A weekly questionnaire, administered in person or by phone was used to monitor adverse events plus changes in health status in all participants. A priori definition of any musculoskeletal or cardiovascular event attributable to testing or training (i.e. inflammatory response in knee joint, cartilage/ligament/muscle tear, fracture, fall, angina, etc.) were considered as adverse events [34].

Pain during a training session that was self-limited and not considered consistent with an injury (see above) was not considered an adverse event but may have resulted in an adjustment of training protocol to accommodate limitations. Protocol deviations or adjustments occurred for both the sham-exercise and the PRT group. The main deviation included changing from a dynamic to an isometric form of training (maximal intensity for PRT and sub-maximal intensity for sham-exercise group) if the dynamic mode was causing pain in the knee joint, reducing the intensity for the PRT group, and/or limiting the range of motion.

### Objectives and Hypothesis

Our objective was to determine the efficacy of PRT as a disease-modifying intervention in women with OA.

We hypothesized that high intensity PRT would decelerate the tibial and femoral cartilage degeneration (i.e. reduce the rate of cartilage loss) in the knee affected most by OA and that high intensity PRT would lead to greater improvements in body composition, physical performance, symptoms and habitual physical activity level, compared to sham-exercise.

### Blinding

All participants were blinded to the investigators' hypothesis as to which was the preferred group. Analysis of the primary outcome (MRI scan of cartilage morphology) was double-blinded at all time points. All baseline and follow-up physical performance and self-report assessments were performed double-blinded; except for follow-up physical performance testing, which was single blinded (participant only). At the final assessment, participants were required to fill-out a Completion Questionnaire without the assessor present. This questionnaire assessed the participants' perception of whether they felt they had been in the "preferred group" to "modify cartilage, increase muscle mass and strength, reduce pain, and improve physical function".

### Assessments

Physician screening was completed initially, followed by an MRI scan. If subjects were eligible following their MRI scan, the remainder of the baseline physical performance testing was completed. Subjects were randomized at the completion of all baseline assessments.

### Primary Outcome: Cartilage Thickness

A 3Tesla MRI scan (Philips Medical Systems, Achieva 3T) of the knee with the most severe clinical signs and symptoms was conducted at the Symbion Clinical Research Imaging Centre at baseline and following the 6-month intervention. OA cartilage morphology assessed via MRI is a reliable and valid technique, particularly for clinical trials where cartilage structure modification is an outcome [35,36]. Depending on the size of the participant's knee, either a SENSE Knee coil (smaller knee) or a SENSE Flex-L coil (larger knee) was used. The same coil was used for pre and post scanning. Prior to the commencement of the scan, a bead was placed on the thigh, halfway between the inguinal groove and the proximal margin of the patella measured anthropometrically by the same observer (AL) in all participants; this marker identified the area for the cross sectional image of the thigh region. The scans included a 3-Dimensional image T1 weighted gradient echo sequence of the tibiofemoral joint (repetition time = 34 ms, echo time = 9 ms, acquisition time = 9 min, flip angle = 25°, slice thickness = 1.4 mm, in-plane resolution = 0.31 mm), and a spin echo sequence of a cross-sectional slice of the thigh region (repetition time = 450 ms, echo time = 10 ms, acquisition time = 2 min, flip angle = 90°, slice thickness = 10 mm, in-plane resolution = 0.47 mm).

**Figure 2:**
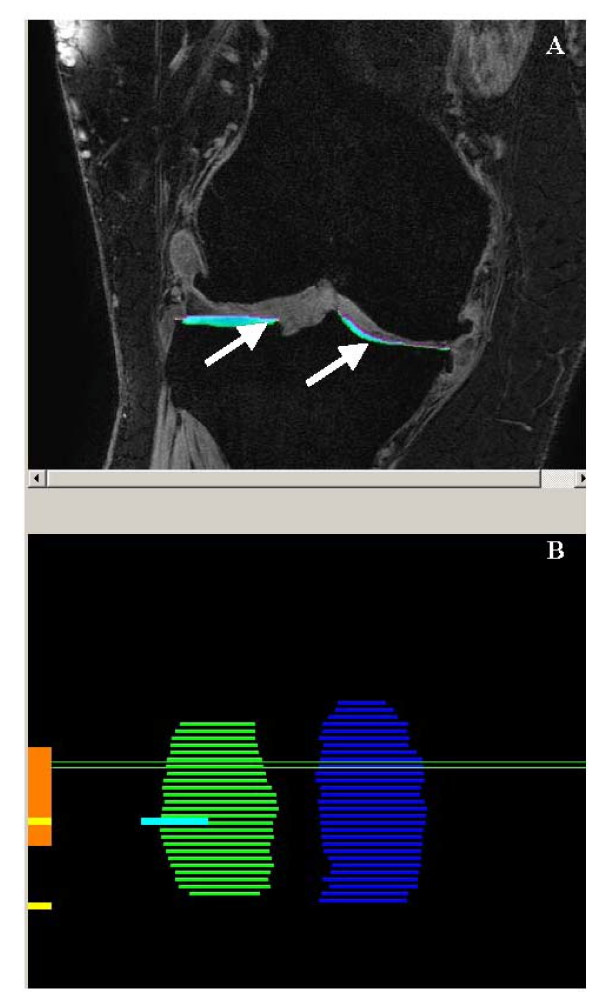
**Part A: Sagittal view of right knee, arrows pointing to segmented (colored areas) areas of tibial cartilage (Medial = right-hand side, Lateral = left-hand side) using Chondrometrics software**. Part B: Medial (blue) and lateral (green) joint surface areas representing the tibial bone covered with articular cartilage.

Blinded measurement of cartilage morphology involved the segmentation of articular cartilage in the medial and lateral tibia, and the central portion of the medial and lateral femur. Any cartilage or bone associated with osteophytes was excluded from segmentation. Chondrometrics software (see Figure 2) (Chondrometrics, Ainring, Germany) was used to segment the cartilage (coefficient of variation (CV) for cartilage thickness: medial tibia = 2.2%, lateral tibia = 2.1%, medial femur = 2.2%, lateral femur = 1.8%; cartilage volume: medial tibia = 3.2%, lateral tibia = 2.3%, medial femur = 3.9%, lateral femur = 1.6%). Datasets were analyzed in pairs, and the person responsible for segmenting each scan (AL) was blinded to the participants ID number and the timepoint of the scan. Quality control of all segmentations was performed by a single person (BV), reviewing all segmented slices of each data set. In addition, automatic quality control procedures were used to exclude mislabelling of medial versus lateral cartilage plates, tibial versus femoral cartilage plates and cartilage versus total subchondral bone contours, the software checking the distance vectors between different plates/contours and a fibular marking. Following cartilage segmentation, computations performed by Chondrometrics software provided outputs for medial and lateral tibial and femoral total subchondral bone area; denuded area (area of subchondral bone eroded, full thickness defect); mean thickness (mean cartilage thickness over total subchondral bone area), thickness covered (mean cartilage thickness over cartilage-covered subchondral bone area), cartilage volume, and VCtAB (volume of cartilage divided by total subchondral bone area).

The outputs for each compartment were analyzed separately; and compartments were also combined, i.e. total tibial cartilage volume (medial + lateral) =

$$\frac{\text{(Total tibial volume at baseline}-\text{Total tibial volume at 6 months})}{\text{Total tibial volume at baseline}}\times 100$$

The same equation applied for total femoral cartilage volume. Total medial compartment volume (medial femoral volume plus the medial tibial volume) was calculated, and the same equation applied for the total lateral compartment volume.

In addition to total compartment analysis, participants were categorized into both geographical OA (medial, lateral, or bi-compartmental) and also, the least and most affected compartment based on MRI Grade classification and osteophytes.

Image J software (Image J software v 1.37, Wayne Rasband, ) was used to calculate muscle and fat volume by a single blinded assessor (CV muscle = 0.16%, fat = 0.2%).

Two blinded physician assessors conducted baseline clinical musculoskeletal system evaluation and MRI interpretation (OA graded according to the MRI correlation of the Modified Outerbridge Classification 1961) at baseline and 6 months independently of each other.

### Secondary Outcomes

#### Dynamic muscle strength

Lower extremity peak strength was assessed using digital K400 Keiser pneumatic resistance machines (Keiser Sports Health Equipments, Inc., Fresno, CA). One repetition maximum (1RM) tests were performed according to de Vos and colleagues [37] unilaterally on knee extension, hip abduction and adduction; and bilaterally on knee flexion, leg press, and plantarflexion. Strength tests were performed twice at baseline approximately 1 week apart, and the higher of the 2 results was recorded as the 1RM. The mean (range) CV for the 9 exercises in this cohort was 13.1 (9.8–21.7) %.

#### Muscle Contraction Velocity

Peak muscle contraction velocity was assessed in a non-fatigued state on a separate day, several days after their final strength test. The test was performed at 20% baseline 1RM on Keiser resistance machines: unilateral knee extension, and bilateral knee flexion and leg press [37].

#### Muscle Endurance

Muscle endurance was assessed after resting from velocity testing. Participants were instructed to perform as many consecutive repetitions as possible at 90% of their baseline 1RM through their full range of motion in correct form. The test was terminated if correct technique was not achieved, a visible pause occurred, or the participant began experiencing significant pain in their knee joint. Number of repetitions, along with mean work, velocity and power were recorded for the first repetition and the last correct repetition. The ratio of last/first repetition power was used as an index of fatigue.

Muscle velocity and endurance were performed during the first training session, with loads based on the higher of the 1RM values obtained. Follow-up endurance testing was conducted using the same 90% 1RM load used during baseline testing.

#### Physical Performance

##### Walking Endurance

The 6-minute walk test, performed according to Guyatt and colleagues [38] was used to assess walking endurance to the nearest 0.1 m. The better of 2 trials 1 week apart was recorded. The CV in this cohort was 3.0 (0.0–13.0) %.

##### Balance

The Chattecx Dynamic Balance System (software version 4.20; Chattecx Corp, Chattanooga Group Inc., Hixson, TN) was used to assess balance. This system allows testing of static balance time and body sway via measurements from the force platform [39]. Three test conditions were performed with eyes open and with eyes closed in a random order and without prior practice: i) narrow bilateral stance on the platform sliding backward and forward at a speed of 8.3 s/cycle in the anterior-posterior direction; ii) narrow bilateral stance on the platform tilting up and down from 0 to ± 2 degrees in the anterior-posterior direction; and iii) unilateral stance of the preferred leg on still platform. Balance was tested for up to 30 seconds per test. A maximum of 3 trials was allowed to complete each test if participants lost their balance (touching hand rails, taking a step off the platform, requiring support from the assessor). If no attempt was successful, the trial with the longest time was recorded and only data from this trial was analyzed. Unilateral stance duration with eyes closed, summated maximum sway in 4 directions (medial, lateral, anterior and posterior), and number of trials needed to complete the 6 conditions were used as individual measures of balance performance.

Overall balance performance was examined using a balance index [40]. The balance index was calculated by summating all anterior-posterior and medio-lateral sway measures and time results respectively.

This index has been shown to be reliable and valid, and is sensitive to change over time with an exercise intervention in older adults [40].

##### Stair climb

Maximal stair climb was used as a proxy for lower extremity power [41]. Two trials were conducted of the 9-step stair climb with 30–60 seconds rest between each trial. The best of the 2 test results was used. Stair power was calculated according to the formula below [41,42]:

$$\text{Power (W)}=\frac{\text{Body Weight (N)}\;\times \text{Height of Stairs (m)}}{\text{Ascent Time }(\text{s})}$$

##### Chair stand

Five chair stand test, performed according to Guralnik and colleagues [43] was used as an index for lower extremity power/balance. Time taken, as well as number of stands completed was recorded. The need for assistance from armrests was also recorded (yes or no) and analyzed as a separate outcome.

### Gait Velocity

Gait analysis was performed using a 10-camera Motion Analysis system (Motion Analysis, Santa Rosa, California) set to sample at 100 Hz. Thirty-eight passive reflective markers were placed bilaterally on standard bony landmarks of the lower and upper body. Participants were asked to walk barefoot without any assistive devices at their self-selected normal and maximal speed for 5 trials. Gait velocity was defined as the mean horizontal velocity of the sacrum marker during 2 full strides and was averaged over 5 trials. The CV in this cohort of the 5 trials was 4.6 (0.7–12.5)%.

### Body Composition

Body mass index was calculated from fasting weight and stretched stature measurements [44]. Waist circumference was measured according to the International Diabetes Federation protocol [45]. Percent body fat and fat-free mass were estimated using bioelectrical impedance (BIA-101: RJL Systems, Detroit, MI) All participants were measured 3 times early in the morning after a 12-hour fast. The CV in this cohort for resistance was 0.03 (0.0–0.1)%. Fat mass and fat-free mass were calculated from the formula developed by Lukaski and colleagues for older adults [46].

### Questionnaires

All questionnaires were interviewer-administered in a private room using visual prompts.

Symptomatology and disability was assessed using the Western Ontario and McMaster Osteoarthritis Index (WOMAC) questionnaire [47]. Habitual physical activity levels were assessed using the Physical Activity Scale for the Elderly (PASE) questionnaire [48] and Harvard Alumni Questionnaire [49]. Depressive symptoms were assessed using the Geriatric Depression Scale (GDS) [50]. Physical self-efficacy was assessed for lifting objects, walking, jogging, climbing stairs, and doing push-ups using the Ewart Self-Efficacy scale [51]. Health-related quality of life was assessed using Version 2 of the Medical Outcome Survey 36-item Short-Form (SF-36) [52]. All questionnaires have been well validated in OA and elderly cohorts.

### Covariates

Covariates identified *a priori *included age, BMI, duration of OA, use of glucosamine and/or chondroitin sulfate, number of chronic diseases. Other covariates will be selected if potential confounders are identified amongst baseline participant characteristics.

### Definitions

Session compliance was defined as the number of sessions attended out of 78 available sessions (up to 1 month extension was given to make up any missed sessions during the 6-month intervention). Training intensity compliance was defined as the difference between the theoretical relative load and the actual relative load for exercise for each session. The theoretical relative load (relative to the most recent 1RM) and the actual relative load was calculated at 4, 8, 12, 16, 20, 24, and 26 weeks. The actual load was then subtracted from the theoretical relative load to get difference in relative loads. Difference should be equal to zero if training intensity compliance was perfect or the results will be negative if actual was greater than theoretical. A one sample t-test was then performed to see whether or not the difference in loads was significantly different than "zero", the ideal value.

Total tonnage per exercise was calculated by summating the training load by the number of repetitions completed for that exercise for the intervention (24 repetitions for 78 sessions (total of 1872 repetitions) per exercise for the PRT group and 16 repetitions for 78 sessions (total of 1248 repetitions) per exercise for the sham-exercise group).

Whole body total tonnage was calculated by summating the total summated loads of each exercises and multiplying that by the total number of repetitions of all exercises.

Dropouts were those participants who did not complete the intervention and did not complete their final assessment (i.e. loss to follow-up). Discontinued subjects were those who did not complete the intervention, but did complete their final assessment and were included in the complete case analysis.

### Sample Size

Reginster and colleagues [53] conducted an RCT of glucosamine vs. placebo in patients with OA, and reported no loss in joint space width (mean -0.06 mm) in the glucosamine group vs. a -0.31 mm joint space loss in the placebo group (p = 0.043). Radiographic joint space loss has been shown to be closely related to reduction in cartilage thickness by MRI, our primary outcome [54]. We conservatively assumed, given the lack of published data, that the protective effect of PRT would be 20% less than that of glucosamine (i.e. 80% rather than 100% reduction in cartilage loss). Therefore, our sample size was estimated from the expected rate of cartilage thinning in sedentary patients with OA of -2.8 ± 2.7% per 6 months [55], which we hypothesized would be unchanged by sham exercise in the controls but would be reduced by 80% to -0.56 ± 2.7% by PRT exercise. The sample size was inflated by 20% to accommodate the anticipated dropout rate over 6 months, based on previous exercise and OA trials in the literature and in our experience. Therefore, the final sample size targeted was 63.

### Randomization

Using a computerized randomization program , a co-investigator uninvolved in participants testing or training randomly allocated participants into 1 of 2 groups. Participants were stratified according to glucosamine and/or chondroitin use (current or within the past 6 months) and Physical Function (Section C; Disability) WOMAC score (< or > 27) in order to equalize these potential confounders between groups. After completion of baseline assessments, participants were given a sealed envelope containing the allocated group (A or B) in accordance with the randomization sequence.

### Statistical analysis

Data were inspected for normality visually and statistically (skewness -1 ≥ 1), and expressed as mean and standard deviation or median and range, as appropriate. Non-normally distributed data were log-transformed prior to use with parametric statistics if possible or used with non-parametric tests if assumptions of normality were not met despite transformation. Our primary analytic strategy was a complete case analysis because of the novelty of our primary outcome. Our secondary sensitivity analysis was intention-to-treat with data imputed via the expectation maximization algorithm (covariates included in the model were group, baseline score, and compliance). Comparisons between groups were made using repeated measures analysis of co-variance (ANCOVA) for both time and group × time interactions, and ANCOVA models for % change scores adjusted for baseline value of each outcome for normally distributed continuous data. Additional covariates considered for inclusion in these models were characteristics at baseline which were different between groups and related to the dependent variable of interest. The Kruskal-Wallace test or Mann-Whitney U test were used for non-normally distributed continuous data. SPSS (Release 13.0 for Windows, 2004, Chicago: SPSS Inc) was used for all data analysis. All *P *values of less than 0.05 were considered statistically significant except for post-hoc comparisons of pairs of variables from significant Kruskal-Wallace models, which were adjusted for multiple comparisons using the method of Bonferroni. Clinical meaningfulness of differences observed was assessed by evaluation of the magnitude of the differences relative to clinical outcomes in the literature, and calculations of effect sizes (Formula 1) adjusted via Hedges bias-corrected effect size for small sample sizes [56]. Effect sizes were interpreted according to Cohen's interpretation of 'trivial' ( < 0.20), 'small' (≥ 0.20 < 0.50), 'moderate' (≥ 0.50 < 0.80), and 'large' (≥ 0.80) effect size [56]. Caution was taken when using Cohen's interpretations as these were originally based on psychological studies. 95% confidence intervals (CIs) for the relative ES were calculated.

$$\begin{array}{cc} \text{Effect Size}=\frac{\Delta \text{ Treatment}-\Delta \text{ Control}}{\text{Pooled SD}} & \left(\text{Formula }1;(56)\right) \end{array}$$

## Discussion and conclusion

Our primary outcome results of cartilage morphology are anticipated in July 2009. This information will provide the first evidence for the efficacy of PRT as a disease-modifying treatment for OA of the knee. Our robustly designed study will be one of very few OA studies that conform to all CONSORT requirements for the reporting of RCTs [57]. In addition, the REACH study will be the first RCT to provide information on the feasibility of using a sham-exercise control group in OA clinical trials.

## Abbreviations

OA: Osteoarthritis; PRT: Progressive Resistance Training; MRI: Magnetic Resonance Imaging; BMI: Body Mass Index; REACH: Resistive Exercise for Arthritic Cartilage Health; ACTR: Australian Clinical Trials Registry; NSW: New South Wales; USA: United States of America; 1RM: 1 Repetition Maximum; RPE: Rating of Perceived Exertion; CV: Coefficient of Variation; ISAK: International Society for the Advancement of Kinathropometry; BIA: Bioelectrical Impedance; WOMAC: Western Ontario and McMaster Osteoarthritis Index; GDS: Geriatric Depression Scale; PASE: Physical Activity Scale for the Elderly; SF-36: 36-item short-form health survey; RCT: Randomized Controlled Trial; ANOVA: Analysis of Variance; ANCOVA: Analysis of Covariance.

## Competing interests

The authors declare that they have no competing interests.

## Authors' contributions

AL, MFS, BV, RMS, MB, and NF participated in the conception and design of the study; AL, NF, and MB participated in recruitment of participants, supervised participant training sessions, and data collection; RS provided radiography reports for all participants; AL conducted the statistical analysis; AL, MFS, and BV were involved in drafting the manuscript or revising it; all authors read, commented, and approved the manuscript.

## Authors' information

AL: B Appl. Sci (Exercise and Sport Science); Discipline Specialist and Research Assistant University of Sydney, Australia.

## Pre-publication history

The pre-publication history for this paper can be accessed here:


